# Trend Analysis of Leukemia Mortality and Years of Life Lost (YLL) from 2004 to 2019 in the Fars Province, Iran

**DOI:** 10.34172/aim.2023.80

**Published:** 2023-10-01

**Authors:** Habibollah Azarbakhsh, Fatemeh Rezaei, Jafar Hassanzadeh, Seyed Parsa Dehghani, Maryam Janfada, Alireza Mirahmadizadeh

**Affiliations:** ^1^Ahvaz Jundishapur University of Medical Sciences, Ahvaz, Iran; ^2^Research Center for Social Determinants of Health, Jahrom University of Medical Sciences, Jahrom, Iran; ^3^Research Center for Health Sciences, Institute of Health, Department of Epidemiology, Shiraz University of Medical Sciences, Shiraz, Iran; ^4^School of Medicine, Shiraz University of Medical Sciences, Shiraz, Iran; ^5^Medical Records, Health Vice-chancellor, Shiraz University of Medical Sciences, Shiraz, Iran; ^6^Non-Communicable Diseases Research Center, Shiraz University of Medical Sciences, Shiraz, Iran

**Keywords:** Iran, Joinpoint regression, Leukemia, Mortality rate, Years of life lost

## Abstract

**Background:**

Although the incidence of leukemia’s is not high, many of these cancers lead to death over a short period. This is a cross-sectional study on leukemia deaths in southern Iran.

**Methods:**

All deaths due to leukemia in the Fars province were obtained from the population-based electronic death registration system (EDRS). Crude and age-standardized mortality rate (ASMR), YLL, and YLL rate data were calculated, and joinpoint regression was used to examine the trend.

**Results:**

Totally, 3141 deaths from leukemia occurred in the Fars province during the study period (2004-2019). Of these, 61.5% (1933 cases) pertained to men. The crude mortality rate was 6.1 (95% CI: 5.8 to 6.4) in men and 3.9 (95% CI: 3.7 to 4.2) in women. Also, ASMR was 6.6 (95% CI: 6.3 to 6.9) and 4.2 (95% CI: 4.0 to 4.4) in men and women, respectively. The total YLLs due to leukemia were 32804 in men and 23064 in women. The joinpoint regression analysis demonstrated that the trend of YLL rate due to premature mortality was stable: the annual percent change (APC) was -1.2% (95% CI: -2.5 to 0.2, *P*=0.090) for males, and -1.0% (95% CI: -2.9 to 0.9, *P*=0.274) for females.

**Conclusion:**

The mortality and YLL due to leukemia had a stable trend. However, this trend has been decreasing or increasing in some age groups. Determining and controlling essential risk factors, especially the environmental factors of leukemia, may reduce its burden in the Fars province.

## Introduction

 Leukemia is a heterogeneous group of hematopoietic malignancies that include four main subtypes described by most cancer registries: acute lymphoblastic leukemia (ALL), acute myeloid leukemia (AML), chronic lymphoblastic leukemia (CLL) and chronic myeloid leukemia (CML).^1^ According to GLOBOCAN, in 2018, leukemia accounted for 437 033 cancer cases and 309 006 cancer deaths, thus becoming the 15^th^ most commonly diagnosed cancer and the 11^th^ leading cause of cancer-related death worldwide. Globally, men have a higher burden of leukemia than women. In 2018, the age-standardized incidence rate was 6.1 per 100 000 for men compared to 4.3 per 100 000 for women, and the mortality rate was 4.3 per 100 000 in men compared to 2.8 per 100 000 for women.^2^ According to the GLOBOCAN Cancer 2020, leukemia accounts for 2.5% of all new cancer cases and 3.1% of all cancer deaths.^3^ The lowest incidence of leukemia pertains to Central and West Africa (less than 3 per 100 000 in men and less than 2 per 100 000 in women), and the highest to North America and Australia/New Zealand (more than 10 per 100 000 men and 7 in 100 000 women). The estimated low incidence in sub-Saharan Africa may be due to the under-diagnosis of the disease in the elderly or very young patients.^4^ Leukemia is the cause of 8% of all cancer cases. It affects all age groups with different prevalence and incidence rates in Iran and the whole world and causes significant mortality and high costs for the diagnosis and treatment process.^5^ Although the overall incidence is low, leukemia is the most common type of childhood cancer, accounting for 30% of all cancers diagnosed in children under 15 years of age.^6^

 Although the incidence of leukemia’s is not high, many of these cancers lead to death over a short period.^7^ Cancer in Iran is the third cause of death after cardiovascular diseases and traffic accidents, accounting for 20% of deaths. Investigating leukemia in further research in Iran indicates the geographical diversity of risk factors in different provinces.^8^ In Iran, registration of incidence rates has shown that leukemia is the seventh most common cancer after skin, breast, stomach, colon, bladder, and prostate cancer.^9^

 The years of life lost (YLL) are an essential criterion for ranking the health status of a society and observing its challenges. According to the World Health Organization report, the value of one year of life is three times more than the gross domestic product (GDP) per capita of any country.^10^ The latest Global Burden of Diseases study showed that the trend of YLL due to leukemia was decreasing worldwide.^11^ In Brazil, between 2001 and 2019, more than 2.2 million YLLs due to leukemia were reported.^12^ In a study conducted in Iran in 2016, leukemia was the third cause of YLL after stomach and lung cancers.^13^ Without understanding the burden of cancer and prioritizing based on it, it will not be possible to implement, monitor, and adequately plan interventions for cancer prevention and control. As a result, this study analyzed the mortality rate and YLL due to leukemia in the Fars province.

## Materials and Methods

 This cross-sectional study was conducted in the Fars province from 2004 to 2019. We extracted all leukemia deaths from the population-based electronic death registration system (EDRS) by age, sex, and year of death and according to ICD-10. The code used in this study was C91-C96. In the population-based EDRS, we used all available sources to detect, record, and collect death-related information.^14^ Repeated deaths, identified based on the father’s name and national identification number, were excluded from the study.

 The total estimated population’of the Fars province was calculated using the primary data of health centers and the population and housing census from 1996 to 2016, taking into account the annual population growth. For standardization, the standard population of 2013 for countries with low and middle income was used.^15^

###  Statistical Analysis

 First, crude and age-standardized mortality rates (ASMR) of leukemia were calculated during the study years according to age and sex groups.

 Then, to calculate YLL, using the standard life table and determining the life expectancy for different age and sex groups, as well as the number of deaths due to leukemia, in each age and sex group, and based on the following relationship, the calculation was made.^16,17^


$$YLL=N\;Ce^{\left(ra\right)}/{\left(\beta +r\right)}^{2}\left[e^{-\left(\beta +r\right)\left(L+a\right)}\;\left[-\left(\beta +r\right)\left(L+a\right)-1\right]-e^{-\left(\beta +r\right)a}\;\left[-\left(\beta +r\right)a-1\right]\right]$$

Where N = number of deaths in an age and gender-specified group; L = life expectancy of death cases in the same age and gender group; r = Discounting Rate, that is 0.03; β = a conventional rate in calculating age value which equals 0.04; C = 0.1658, which is an adjusted, constant value; a = the age when death occurred; and e = a constant value considered as 2.71.

 First, the YLL was calculated based on 18 age groups: 0–4, 5–9, 10–14, etc., up to 85 years, and then based on age groups 0–4, 5–14, 15–29, 30–44, 45–59, 60–69, 70–79 and over 80 years are shown in a figure.

 The number of YLLs due to premature death caused by leukemia was analyzed using the YLL template of 2015, World Health Organization (WHO), in Excel version 2016.

 Joinpoint regression based on the log-linear transformation was used to evaluate the trend of crude and age-standardized mortality and YLL rates for different years. Joinpoint regression analysis demonstrates changing trends over successive segments of time and the increase or decrease of each part. The resulting line segment between joinpoints is described by the annual percent change (APC) based on the line segment’s slope and the average annual percent change (AAPC). Unlike pitch-based linear regression, log-linear regression is based on the APC. (i.e. the rates change at a constant percent per year), and can also be used to study trends across scales.^18^ The joinpoint regression model can be written as follows:


$$Log\left(yi\right)=\beta 0+\beta 1ti+Y1{\left(ti-Ti\right)}^{+}+...+Yk{\left(ti-Tk\right)}^{+}+\varepsilon i,\;i=1,.......,n$$

Where ti indicates the time points (2004, 2005… 2019), yi represents the YLL rates, K shows the number of change points, β0, β1 and γ1… γk indicate the regression coefficients, and εi is the model error term. By fitting the joinpoint regression, we can calculate the APC in rates between the estimated change points. To do this, the log transform of the model is utilized. APC = 100 × (exp (β1 + 𝑌1* I (𝑡𝑖 -𝑇𝑖) + … + 𝑌𝑘*I (𝑡𝑖 -𝑇𝑘)) -1).^19^

 In our analysis, X_i _represented the years between 2004 and 2019, and y_i_ is the annual burn YLL rate.

 We used constant variance (homoscedasticity) and uncorrelated in our analysis. The Joinpoint Regression Program 4.9.1.0 was used to perform the analysis for the trend.^20^

## Results

 During the 16-year study period (2004–2019), 3141 deaths from leukemia occurred in the Fars province, which constituted 10.1% (3141/30 936) of all cancer deaths in this period. Of these, 61.5% (1933 cases) occurred in men, and 18.3% (574 cases) were in the age group of 45-59 years.

 As shown in Table 1, the crude mortality rate due to leukemia had a stable trend in men, with 6.3 per 100 000 population in 2004 to 5.8 per 100 000 population in 2019 (AAPC = 0.5%; 95% CI: -0.4 to 1.4, *P* for trend = 0.227); also in women, it ranged from 3.8 per 100 000 population in 2004 to 3.7 per 100 000 population in 2019, which is a stable trend (AAPC = 0.5%; 95% CI: -1.5 to 2.5, *P* for trend = 0.624). The ASMR had a steady tendency in men, from 7.7 in 2004 to 6.3 per 100 000 in 2019. (AAPC = -0.6%; 95% CI: -1.5 to 2.0, *P* for trend = 0.132). In women, it had a stable trend, from 5.2 in 2004 to 3.7 (per 100 000) in 2019 (AAPC = -2.2%; 95% CI: -3.3 to 0.7, *P* for trend = 0.170) (Table 1).

**Table 1:** Crude and Age-Standardized Mortality Rate (Per 100 000 Population) and Years of Life Lost due to Leukemia by Gender and Year in the Fars Province During 2004–2019

**Year**	**No. of Death**		**Crude Mortality Rate**		**ASR (95%CI)**		**YLL**			
							**No.**		**(per 1000)**	
	**Male**	**Female**	**Male**	**Female**	**Male**	**Female**	**Male**	**Female**	**Male**	**Female**
2004	117	67	6.3	3.8	7.7 (6.6-8.8)	5.2 (4.3-6.1)	2205	1331	1.18 (1.13-1.23)	0.74 (0.70-0.79)
2005	101	77	5.5	4.3	6.5 (5.5-7.5)	5.3 (4.3-6.3)	1953	1603	1.05 (1.00-1.10)	0.90 (0.85-0.94)
2006	99	53	5.4	2.9	6.4 (5.4-7.4)	3.6 (2.8-4.4)	1921	1086	1.03 (0.99-1.08)	0.60 (0.56-0.63)
2007	109	83	5.8	4.5	6.5 (5.4-7.6)	5.2 (4.2-6.2)	2000	1890	1.07 (1.02-1.11)	1.03 (0.98-1.08)
2008	122	64	6.5	3.5	7.2 (6.1-8.3)	3.7 (2.8-4.5)	2261	1317	1.19 (1.15-1.24)	0.71 (0.67-0.75)
2009	117	73	6.1	3.9	6.8 (5.7-7.9)	4.5 (3.6-5.4)	2042	1437	1.07 (1.02-1.11)	0.76 (0.72-0.80)
2010	122	72	6.3	3.8	6.9 (5.8-8.0)	4.1 (3.2-5.0)	2134	1430	1.10 (1.06-1.15)	0.75 (0.71-0.79)
2011	113	61	5.8	3.2	5.9 (4.8-7.0)	3.3 (2.5-4.1)	1893	1287	0.97 (0.93-1.01)	0.66 (0.63-0.70)
2012	116	97	5.9	5.0	6.3 (5.3-7.3)	5.1 (4.1-6.1)	1566	1555	0.79 (0.75-0.83)	0.79 (0.76-0.84)
2013	139	82	7.0	4.2	7.2 (6.1-8.3)	4.2 (3.3-5.1)	2414	1468	1.20 (1.16-1.25)	0.74 (0.70-0.78)
2014	140	77	6.9	3.8	7.0 (5.9-8.1)	4.3 (3.4-5.2)	2534	1548	1.25 (1.20-1.30)	0.77 (0.74-0.81)
2015	115	86	5.6	4.3	5.7 (4.7-6.7)	4.1 (3.2-5.0)	1872	1623	0.91 (0.87-0.95)	0.80 (0.76-0.84)
2016	135	56	6.5	2.8	6.6 (5.5-7.7)	2.8 (2.1-3.5)	2164	1010	1.04 (0.99-1.08)	0.49 (0.46-0.52)
2017	131	86	6.3	4.2	6.2 (5.1-7.3)	4.2 (3.3-5.1)	1957	1603	0.94 (0.89-0.98)	0.79 (0.75-0.83)
2018	135	99	6.5	4.9	6.6 (5.5-7.7)	4.8 (3.8-5.8)	2103	1602	1.00 (0.96-1.05)	0.78 (0.75-0.82)
2019	122	75	5.8	3.7	6.3 (5.3-7.3)	3.7 (2.9-4.5)	1785	1274	0.84 (0.80-0.88)	0.62 (0.59-0.65)
Total	1933	1208	6.1	3.9	6.6 (6.3-6.9)	4.2 (4.0-4.4)	32804	23064	1.04 (1.03-1.05)	0.74 (0.73-0.75)
*P* value	—	—	0.227	0.624	0.132	0.032	—	—	0.090	0.274

YLL, years of Life Lost; ASR, age-standardized rate.

 The total YLLs due to leukemia during the 16-year study period were 32 804 (1.04 per 1000 people) in men, 23 064 (0.74 per 1000 people) in women, and 55 868 (0.89 per 1000 people) in both sexes. (Male/female ratio, 1.42) (Table 1). The average YLL due to leukemia was 16.97 in men, 19.09 in women, and 17.78 in both sexes.

**Figure 1:**
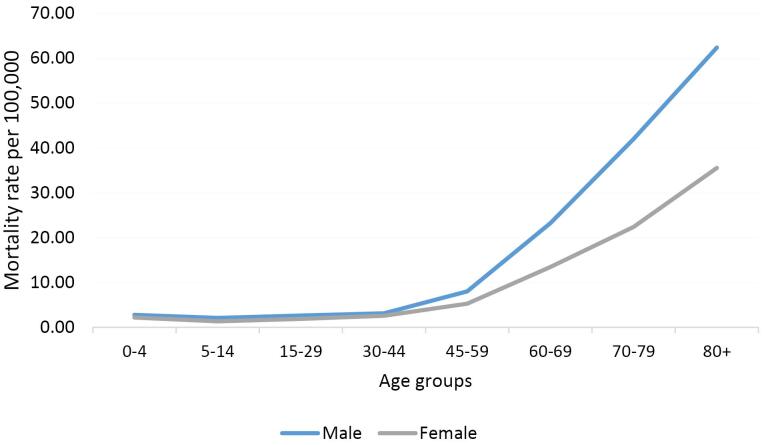
Mortality Rate Due to Leukemia by Gender and Age Groups

 The highest and lowest mortality rates in both sexes pertained to the groups aged over 80 and between 5 to 14 years, respectively (Figure 1).

###  Temporal Trends of Leukemia Mortality by Age Groups

 In the 0–44 age group, the leukemia mortality rate manifested stable trends in men (AAPC = -2.7%, 95% CI -6 to 0.8, *P* = 0.115) but decreasing trends in women (AAPC = -2.8%, 95% CI: -5.2 to -0.3, *P* = 0.033).

 In the 45–59 age group, there were decreasing trends in men (AAPC = -4.4%, 95% CI: -8.1 to -0.5, *P* = 0.030) but stable trends in women (AAPC = -1.6%, 95% CI -5.0 to 1.9, *P* = 0.340).

 In the 60–74 age group, there were stable trends in men (AAPC = 0.8%, 95% CI: -0.8 to 2.5, *P* = 0.310) and women (AAPC = -0.3%, 95% CI: -3.6 to 3.0, *P* = 0.840).

 In the + 75 age group, there were increasing trends in men (AAPC = 2.7%, 95% CI: 0.4 to 5.0, *P* = 0.023) but stable trends in women (AAPC = 2.0%, 95% CI: -4.5 to 8.8, *P* = 0.533).

**Figure 2:**
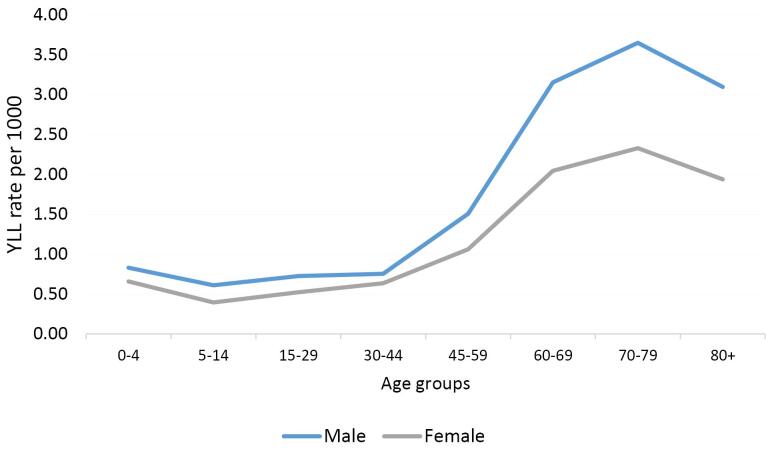
Years of Life Lost Rate Due to Leukemia by Gender and Age Groups

 The highest and lowest YLL rates in both sexes pertained to the age groups of 70–79 years and 5–14 years, respectively (Figure 2).

**Figure 3:**
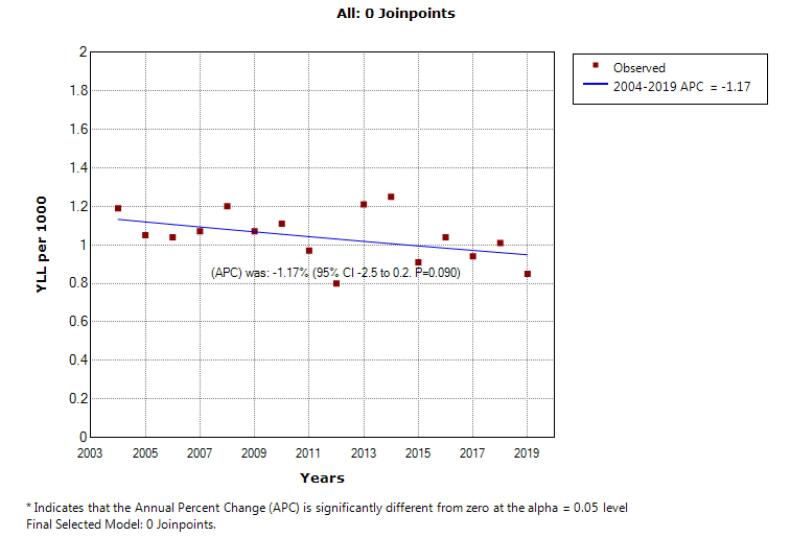
Trend of Years of Life Lost Due to Leukemia in Women During the Years 2004–2019

**Figure 4:**
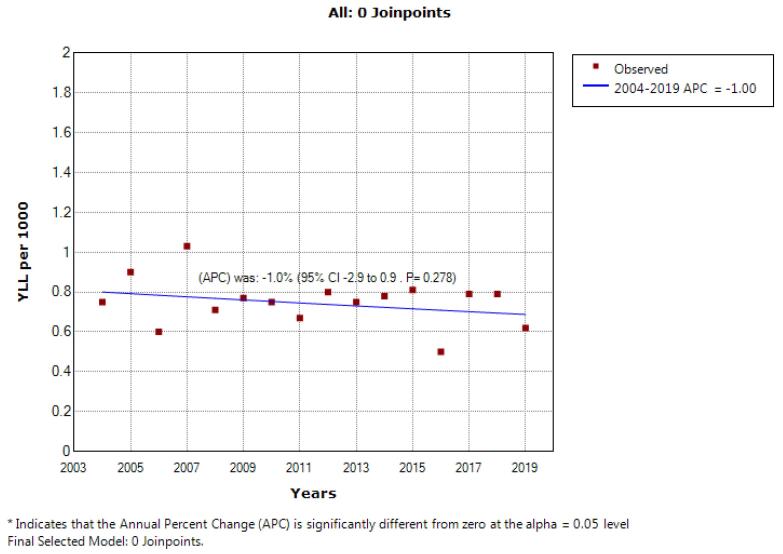
Trend of Years of Life Lost Due to Leukemia in Women During the Years 2004–2019

 According to the joinpoint regression analysis, the 16-year trend of YLL rate due to premature mortality was stable: the APC was -1.2% (95% CI: -2.5 to 0.2, *P* = 0.090) for males, -1.0% (95% CI: -2.9 to 0.9, *P* = 0.278) for females, but decreasing for both sexes: APC -1.1% (95% CI: -2.2 to -0.1, *P* = 0.048). The model did not show any joinpoint; hence, the AAPC was equal to the APC (Figures 3 and 4).

## Discussion

 This study investigated the mortality rate trend and YLL due to leukemia in 16 years from 2004 to 2019 in southern Iran. In this period, the total number of YLLs due to leukemia in the entire study population was 0.89 per 1000, which was higher in men than in women (1.04 per 1000 vs. 0.74 per 1000). However, the average number of YLL due to leukemia was higher in women than men (19.09 vs. 16.97 years). In 2004, the YLL rate was 1.18 per 1000 in men, and 0.74 per 1000 in women. In 2019, the rate of YLL decreased to 0.84 per 1000 in men and 0.62 per 1000 in women.

 Although this decreasing trend was insignificant in men and women, it was significant in general (APC = -1.2, *P* value = 0.090) for men; APC = -1.0, *P* value = 0.274 for women; and APC = -1.1, *P* value = 0.048 overall). In age groups, different changes were observed in the trend of mortality in men and women, such that in the age group of 0–44 years, this trend was constant in men, but decreased in women. In the age group of 45-59 years, this trend decreased in men but was stable in women. In the age group over 75 years, the mortality trend due to leukemia was increasing in men, but decreasing in women. In the age group of 60-74, this trend was constant in both sexes.

 One study showed that on the global scale, the trend of ASMR was decreasing from 1990 to 2017. In recent decades, there have been significant changes in leukemia mortality worldwide, such that mortality has decreased in all age groups. According to the WHO statistics, the death rate from this cancer among all age and sex groups is decreasing in countries such as France, Italy, Great Britain, and Japan.^21^ Also, the death and morbidity of leukemia have decreased in most regions and countries with a high socio-demographic index (SDI), such as Bahrain, Finland, and Austria.^11^

 Although leukemia is diagnosed at a younger age in low- and middle-income countries, the survival interval of leukemia has gradually decreased in most countries.^22,23^ The 2019 GBD study reported that the age-standardized rate (ASR) of death and disability-adjusted life years (DALYs) attributable to four major leukemia subtypes decreased from 1990 to 2017.^24^ In the present study, a decreasing trend of age-standardized mortality was observed from 2004 to 2019, and the ASR of death in 2004 was equal to 7.7 in men and 5.2 in women, which in 2019 was reduced to 6.3 in men and 3.7 in women. The decrease in the death rate and YLL of leukemia is most likely due to the new methods and strategies for diagnosing and treating leukemia. In our study, similar to other studies, the burden of leukemia was greater in men than women.^11,24,25^ In other studies, a relationship between leukemia and smoking has been established.^26,27^ It has been shown that the higher burden of leukemia in male patients compared to female patients may be due to exposure to smoking and other high-risk occupational and environmental factors.^28^ In addition, a national study in the United States found that leukemia deaths decreased in states where smoking rates decreased but remained unchanged in states where smoking prevalence remained relatively constant.^29^

 In a study in Europe, it was shown that the trend of mortality due to leukemia from the years 1970 to 2009 decreased in most European countries, and the age group of 0-14 witnessed the most significant decrease (AAPC = -3.7%) for men and (-3.8%) women. Also, in the age group of 15–44 years, AAPC was -2% for both sexes. In the rest of the age groups, the mortality trend caused by leukemia was decreasing.^30^ The findings of this study in some age groups align with ours and contradict the results of our research in some other age groups.

 Different trends in leukemia mortality in later age groups are also attributed to the different histotype compositions of leukemia at different ages: ALL is the most common type in children and young adults, while CLL prevails in adulthood.^30^

 In the past decades, significant advances have been made in the treatment of leukemia and the prognosis related to its treatment, which has led to a significant change in the survival pattern of leukemia patients. In recent decades, very good treatments for leukemia have been developed and improved, such that the 5-year survival rate for children and adolescents is reported to be 84%.^31^ It should be noted that the disease burden is different across regions and countries. Demographic factors and socioeconomic status affect the disease burden.^32^ It has been shown that the apparent downward trend in leukemia death in areas with high SDI and regions with high income can be due to adequate medical resources and the health care system.^11,33^

 The decreasing trend in age-standardized mortality and YLL observed in this study largely depends on the development of health care in Iran. The comprehensive coverage of primary health care in the villages of Iran created a revolution in population health indicators and reduced inequalities.^34^ Thus, in Iran, primary health care covers a large population in rural areas, and health facilities available to villagers are inexpensive. In this regard, insurance services have been provided to villagers as well as city residents without health insurance. According to the family physician program and rural insurance in Iran, the existing primary health care network is an essential asset in facilitating the implementation of the family physician program in rural areas,^35^ which can significantly improve people’s health.

 In the 0–44 age group, the leukemia mortality rate had a stable trend in men (AAPC = -2.7%, 95% CI: -6 to 0.8, *P* = 0.115) but a decreasing trend in women (AAPC = -2.8%, 95% CI: -5.2 to -0.3, *P* = 0.033). Random error may be the reason for this difference between men and women.

 A limitation of the present study was that YLL was not evaluated throughout Iran due to the unavailability of the necessary data. This study was of high quality and with a strong study design, large sample size, and extensive time period of data analysis.

## Conclusion

 The trend of mortality and YLL due to leukemia in the study period had a relative decrease, which was not statistically significant and was almost constant. However, this trend was decreasing or increasing in some age groups. Determining and controlling important risk factors, especially the environmental factors of leukemia may reduce its burden in the Fars province. In addition, the use of modern and efficient leukemia treatment methods can prevent a significant number of deaths. Analytical studies are recommended to obtain the causal relationship and solve the problems related to the disease.
